# Collagen Based Materials in Cosmetic Applications: A Review

**DOI:** 10.3390/ma13194217

**Published:** 2020-09-23

**Authors:** Alina Sionkowska, Katarzyna Adamiak, Katarzyna Musiał, Magdalena Gadomska

**Affiliations:** 1 Department of Biomaterials and Cosmetics Chemistry, Faculty of Chemistry, Nicolaus Copernicus University in Torun, Gagarin 7 street, 87-100 Torun, Poland; kadamiak@wellu.eu (K.A.); musialk.97@gmail.com (K.M.); 291013@stud.umk.pl (M.G.); 2 WellU sp.z.o.o, Wielkopolska 280 street, 81-531 Gdynia, Poland

**Keywords:** collagen, cosmetics, skin, peptides

## Abstract

This review provides a report on properties and recent advances in the application of collagen in cosmetics. Collagen is a structural protein found in animal organisms where it provides for the fundamental structural support. Most commonly it is extracted from mammalian and fish skin. Collagen has attracted significant academic interest as well as the attention of the cosmetic industry due to its interesting properties that include being a natural humectant and moisturizer for the skin. This review paper covers the biosynthesis of collagen, the sources of collagen used in the cosmetic industry, and the role played by this protein in cosmetics. Future aspects regarding applications of collagen-based materials in cosmetics have also been mentioned.

## 1. Introduction

Collagen as a structural protein constitutes a large part of the connective tissue, particularly in bones, tendons, joints, and skin [1]. The primary structure of collagen consists of amino acids, mostly by glycine (33%), proline, and hydroxyproline (22%). The secondary structure is formed of amino acids α chains bundled up into helix with three amino acids per turn, which are sprained around each other and form a tight tertiary structure. Fundamental collagen structure–quaternary structure pertains to the superhelix. Thus far 29 types of collagen have been discovered. [2]. The differentiation of the collagen nature is a fortiori determined by the existence of various α chains, isoforms of the particles, and supramolecular structures of each collagen type. The diversity of collagen kinds is also caused by differences in the expression of genes involved in protein biosynthesis. Furthermore, posttranslational modifications of collagens also have a significant influence on collagen diversity [3]. When characterizing collagens, we primarily defined their affiliation to a particular group to which they were assigned based on their complexity and structural diversity, presence of non-helical fragments, functions and capability of assembling supramolecular structures [3]. The major types of collagen include: type I collagen (found in skin, tendon, and bone tissue), type II (found in cartilage), and type III (found in skin and vasculature). Collagen has a broad spectrum of applications. It is widely used in the cosmetic, pharmaceutical, medical, and food industry because of its high biocompatibility, non-toxicity, and biodegradability [1,2,3,4]. This particular review will focus on the cosmetic application of collagen. The content of collagen in the skin decreases with age—that is the main reason why cosmetic companies compete to develop different, new methods of topical usage of collagen.

This review aims to summarize the existing knowledge about the sources of collagen for cosmetic applications, the methods of its extraction, and further possibilities of collagen modification for cosmetic uses.

## 2. The Biosynthesis of Collagen

**Figure 1:**
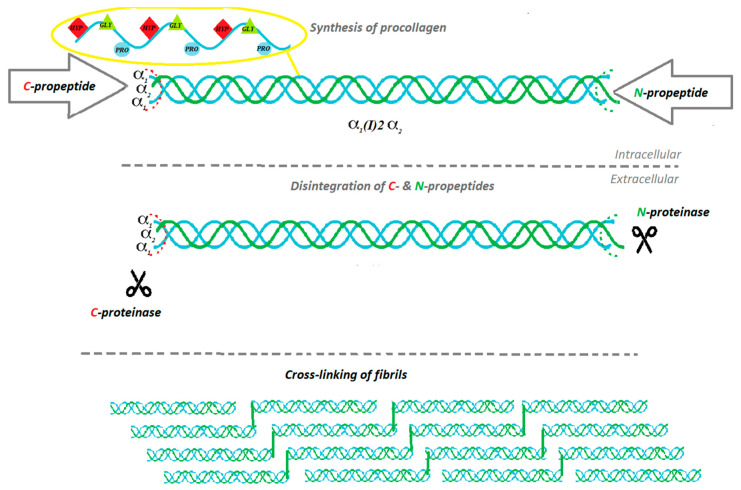
Biosynthesis and processing of collagen type I.

**Figure 2:**
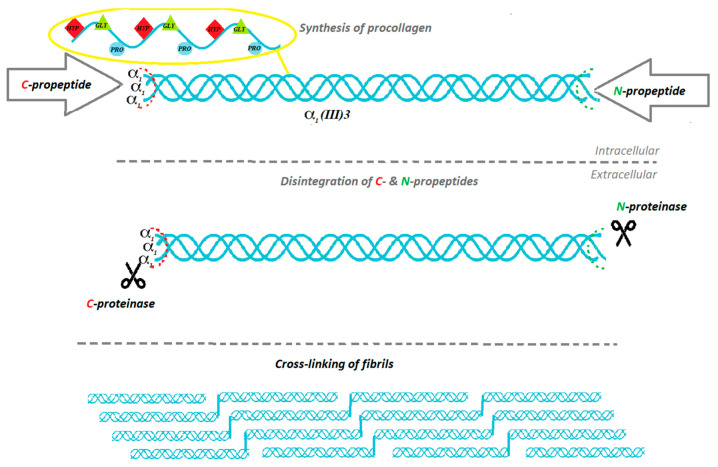
Biosynthesis and processing of collagen type III.

Collagen is the main component that forms the skin. Organism synthesizes it as a procollagen from fibroblasts and later, transforms procollagen into the collagen particle, with 85–90% comprising type I of collagen, and the remaining 10–15% pertains to type III of collagen [5]. The biosynthesis of proteins is to a high extent similar, but biosynthesis of collagen has its unique characteristics, such as precursor form (procollagen), which fulfills significant functions, and a few infrequent posttranslational modifications which appear after the congregation of amino acids into the three peptide chains. It is crucial for the structural characteristics of collagen [6]. The biosynthesis of collagen is a multistage process, which requires numerous biochemical factors and events occurring in proper conditions [7] (see Figure 1 and Figure 2).

Each type of collagen has a different initiative stage of the synthesis process due to encoding different α-chain genes in the three-chain combination. The common steps of biosynthesis include intracellular events and triple-helix formation, post-translational modification, enzymatic glycosylation and lysyl hydroxylation, proteolytic cleavage of procollagen, extracellular supramolecular collection, and natural cross-linking [7]. In the first step, pre-procollagen is processed into procollagen by removal of the signal peptide [7]. The process starts at N-terminus while the creation of triple-helix starts at C-terminus [8]. Some proteins are preventing α chains from folding: prolyl 4-hydroxylate, protein disulfide isomerate, heat shock protein 47, a homologue of heat shock protein 70, and various peptidyl-prolyl cis-trans isomerases [9]. The placement of the pro-α-chains called registration is performed by the C-telo-peptides. Once that happens, the triple helix expands in a zipper-like way from the C- to the N-terminus [10]. Hsp47 prevents the premature cumulation of procollagen [9,11,12]. When the procollagen reaches the Golgi apparatus, Hsp47 detaches. Presumably, it is caused by pH change [7]. Post-translational modification of collagen enfolds hydroxylation and glycosylation [7]. The molecular signage of collagen is hydroxyproline: nearly 10% of all collagen amino acids are represented by hydroxyproline. Prolyl hydroxylation is performed due to the catalytic activity of enzyme propyl 4-hydroxylase. Hydroxyproline occurs at the Gly-X-Y repeatable formations on the Y position. Collagen also contains hydroxylysine and subsequent O-glycosidic bonds that constitute valid modulators of the fibrillogenesis process [13]. When the triple helix is properly folded, the procollagen trimer is released. The stabilization of properly composed procollagen is provided by Hsp47, whereas protein disulfide isomerase prevents procollagen from eliding the endoplasmic reticulum non-folded [14,15]. Afterwards, specific enzymes proteolytically obviate the N- and C-propeptides [16]. Collagen triple helices, also known as tropocollagen, are forming supramolecular units. There are two possible models of fibril formation: the first one, given that fibril formation is taking place inside the specific carriers: the Golgi-to-plasma membrane carriers (GPCs); the second model, provided that formation of the collagen fibrils takes place on the fibroblasts in the cytoplasmic membrane. The process of fibrillogenesis is possible due to fibronectin and integrins. A fibril network is created, serving as a matrix for the subsequent collagen fibril formation [17,18]. At the later stage, molecular packing of collagen units takes place: microfilaments, fibrils, and finally mature collagen filament.

## 3. Sources of Collagen for Cosmetic Use

Collagen is a primary component in many cosmetic formulations due to the fact that it is a natural humectant and moisturizer. The cosmetic industry is continuously seeking for innovative and effective products, making the collagen source a significant matter or study [19]. Origin of collagen may vary from bovine and porcine by-products to marine sources. Bovine and porcine collagen is associated with the risk of zoonotic diseases such as TSE (transmissible spongiform encephalopathy), BSE (bovine spongiform encephalopathy) and FMD (Foot and Mouth Disease). In contrast, marine collagen constitutes a crucial disjunction to other animal sources of this protein [19]. The potential of marine collagen has been discovered approximately 70 years ago during the case study of sponges [20,21]. The research contributed to learning about the structural and physicochemical features of the marine origin collagen. Nonetheless, the first extensive studies of the collagenous structure were performed on the species of *Chondrosia reniformis* and *Ircinia* and focused only on one form of collagen: intracellular type [22,23,24]. Following studies aimed to determine the morphology description of diverse collagen types: ICC, InSc, and SIC. The forms of collagen that had been examined are derived from *Althaea cannabina* and *Staphylococcus carnosus* species. Collagen originating from jellyfish, specifically *R. pulmo* species, represents similar biological activity to the human-like mammalian type I collagen. Research has shown that some of the human receptors are capable of identifying collagen originating from jellyfish. That indicates a similar response concerning cell adhesion, proliferation, and migration like in the jellyfish collagen [25]. Collagen content in jellyfish constitutes approximately 60% [26]. Some research showed that the highest collagen recovery index was found in species such as *Rhopilema asamushi*, *Stomolophus meleagris*, *Catostylus tagi*, and *Rhizostoma pulmo* [25,27,28]. Due to tests results, amino acid content was comparable to vertebrate collagen, nonetheless, a significantly lower content of hydroxyproline was observed. It indicated relatively low denaturation temperatures that were within the limits of 26 and 29.9 °C. Other species of jellyfish contain similar to vertebrate collagen IV and V [29,30]. *S. meleagris* species represent collagen similar to vertebrate collagen type II [31]. The other skin of cuttlefish provides another collagen source for cosmetic use. *Sepia lycidas* species contain 2% to 35% collagen of the lyophilized dry weight [32], whereas the octopus *Callistoctopus arakawai* comprises 10.4% and 62.9% of lyophilized dry collagen weight [33]. Skin from *Illex argentinus* contain approximately 35.6% of collagen [34]. From marine sources of collagen, the fish belong to the most valuable source of collagen. The main reason is that 75% of a fish weight constitutes collagen content [35]. Fish skin are chosen mainly for the purpose of obtaining type I of collagen [36,37,38,39]. The main sources of fish collagen are skins, bones, heads, scales, fins, and entrails [40]. Collagen type II is also found in fish cartilage [40]. The research has shown that fish subjected to a very restrictive diet produce more collagen in comparison to well-nourished fish. Collagen properties depend on the age of the organism. Solubility has a tendency to decrease with time because of a higher number of crosslinkers in older animals [41]. According to research the highest content of collagen is found in silver carp, brown-backed toadfish, codfish, and tilapia [36,42,43,44]. Cosmetic formulations based on marine origin collagen may vary in their properties and composition, depending on species and organism’s age. It is important to determine the properties of specific collagen origin in order to choose the proper one for the formulation [19].

## 4. Collagen Extraction Methods for Cosmetic Use

Depending on the collagen origin, various techniques have been proposed to extract collagen macromolecules. Yet, it is possible to define a comprehensive methodology to isolate collagen from fish by-products using three crucial steps: preparation, extraction, and recovery. Process of collagen extraction requires using one of the three main methods which characterize the production of acid solubilized collagen, neutral salt solubilized collagen, and pepsin solubilized collagen [45]. Properly prepared collagen material derived from young animals can be extracted by neutral salt solutions because of the low number of crosslinked bonds [46]. The material is then purified by processes that include dialysis, precipitation, and centrifugation. Whereas when it comes to the material from older organisms, which contains a higher number of crosslinked bonds making the collagen material less soluble in water, the acidic extraction should be performed. This kind of extraction is way more efficient. Exemplary solvents that can be used in this method are acetic acid, hydrochloric acid, and lactic acid. The fact that the collagen triple helix is resistant to proteases like pepsin and chymotrypsin should also be taken into account [47,48]. During fish processing, wastes such as skin, bones, and scales are generated in approximately 50–70% of the used material [38]. The mentioned materials are rich in collagen and have gained significant attention as a collagen source. The highest potential in cosmetics seems to be exhibited by collagen extracted from fish skin. This kind of collagen extraction is based on several steps: firstly, the skin is minced and mixed with the alkaline solution, then stirred for 24 h. The aim is to remove the non-collagenous protein. The next step is getting rid of the mechanical impurities by straining through a coarse sieve as many times as it is required. The alternating step is the homogenization with the proper acidic solution, then stirring it for another 24 h. Next is the centrifugation process and collecting the supernatant. The residue must undergo the process of extraction once more with the proper acidic solution. The assembled supernatant constitutes the acid-soluble collagen. The material prepared this way can be approached in two ways. First is a repeated homogenization, stirring for 24 h and application of pepsin, then stirring for 24 h, centrifugation, and collecting the supernatant. Then the alkaline solution has to be applied and the stirring process has to be conducted again. A further step is suspending the precipitate in the right buffer to accomplish the proper pH value, which should equal to 7.4. The prepared sample has to be dialyzed against the same buffer and centrifugated. If the pure collagen powder is the final product, the sample must undergo a drying process, for example, lyophilization. The second way of preparing the collagen is a transition from the primarily collected supernatant directly to the step of adding the alkaline solution. This method requires less time, but it is the enzymatic method that is considered more efficient. The method of obtaining collagen material should be tailored based on its source and destination [49].

## 5. Types of Collagen in Cosmetics

Skin consists of tissue built mainly by I, III, and V type of collagen. The dominant type of collagen in the skin is the type I [50]. As shown by research, the marine collagen is abundant in this type of collagen [19]. Thus, the marine collagen is the most desirable collagen source in the cosmetic field. Examination of collagen obtained from tilapia skin showed that as well as acid-soluble collagen (ASC) and pepsin-soluble collagen (PSC) have typical type I collagen features. The ASC denaturation temperature was 36.1 °C, but for PSC it was 34.4 °C [51]. Whereas isolated acid-soluble collagen from the skin of silver carp (*Hypophthalmichthys molitrix*) also comprised type I collagen as shown by SDS-PAGE patterns [52,53], with denaturation temperature established at around 29 °C for ASC. Column chromatography indicated three α chains: α1, α2 and α3 [52]. The collagen content of Atlantic salmon (*Salmo salar* L.) demonstrated types I and V [54]. Circular dichroism (CD) stated the denaturation temperature for salmon collagen at 27 °C [19]. Research on collagen content in cod also revealed the presence of I and V type of collagen [55]. Prosecuted tests on bigeye snapper (*Priacanthus tayenus*) tissues (skin and bone) unveiled two various α chains: α1, α2 as type I collagen. Electrophoretic patterns derived from skin and bone of bigeye snapper were significantly similar [38]. Both ASC and PSC derived from hybrid sturgeon contain collagen type 1, which was confirmed by SDS-Page and FTIR. The ASC denaturation temperature was oscillating at 26.8 °C and at 26.5 °C for PSC, as measured by circular dichroism (CD) and differential scanning calorimetry (DSC) [56]. Native non-denatured collagen is highly desired in cosmetic and biomedical applications. However, fish collagen has its limitations when applied in emulsions that are prepared with the water and oil heating phases, because of the low temperature of denaturation. In several cosmetic formulations hydrolyzed collagen is used, as it still exhibits collagen’s moisturizing properties but at the same time can be applied in emulsions with a high temperature of emulsification.

## 6. Collagen Role in Cosmetics

**Figure 3:**
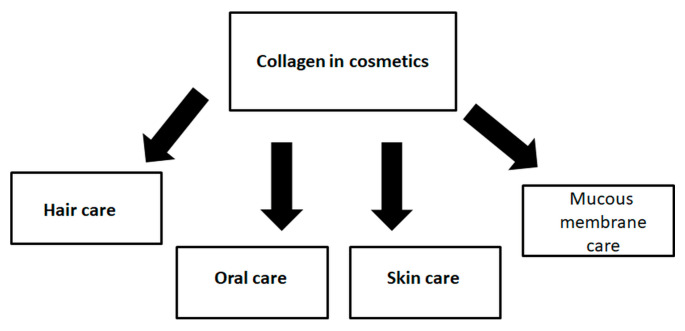
The application of collagen in cosmetics.

Collagen represents one of the main constituents of cosmetic formulations because of its moisturizing, regenerating, and film-forming properties. Excellent ability to bind water helps to maintain proper water content in the skin during the day. The skin is moisturized and softened. Aside from being a natural humectant, collagen’s film-forming properties reduce transepidermal water loss (TEWL). Peptide occlusion prevents skin and hair damage caused by mechanical impairments. Moreover, occlusion makes skin more radiant, illuminated, and smooth [57,58]. Research has shown that collagen accelerates wound healing and helps tissue to regenerate [59,60,61,62,63,64]. Therefore, collagen is widely used in the cosmetic field. It can be used both for skin and hair care. The cosmetic potential of collagen is shown in Figure 3.

Collagen fillers are also used in aesthetic medicine. The subcutaneous injection of soluble collagen improves the quality and density of the skin, repairing its dermatological defects [63]. Collagen fillers are used to remove the signs of ageing. The extender should be biocompatible, non-allergic, easy to remove, and biodegradable in time. Before the procedure, allergy tests must be conducted. Collagen fillers are widely used because of several reasons: they are nontoxic, natural, biodegradable, and the effects are reproducible [65,66,67,68].

Collagen is the main component of several hydrogels which can be used as a so-called “beauty mask”. This kind of cosmetic should restore skin elasticity and promote anti-ageing performance [69]. Many polymers of natural origin can also play a significant role in cosmetic formulations, acting as a thickening agent. Even though collagen itself can also be used as a thickening agent, the high price of native collagen prevents such choice. Instead, it is gelatin—a much cheaper, denatured form of collagen—that is usually chosen as a thickening agent [70].

## 7. Hydrolyzed Collagen for Cosmetic Purposes

As it was mentioned above, when collagen is undergoing maturation, it becomes less soluble in water and acidic pH due to the increasing number of mature crosslinks. For this reason, in several cosmetic formulations, mainly hydrolyzed collagen is used. Short polypeptides and small peptides which derived from collagen are well soluble in water and it is very easy to incorporate such hydrophilic molecules into the cosmetic formulation [70]. Moreover, short polypeptides and small peptides can penetrate into deeper levels of the skin, making it possible to regenerate skin properties. For the preparation of collagen hydrolysates, the fish waste was studied as a raw material [71]. Enzymatic hydrolysis was selected for recovering these by-products with high value-added. The moisturizing properties of hydrolyzed collagen in deeper levels of the skin are very high and may give a very good effect on the looks of the skin [72,73,74]. In its hydrolyzed form, collagen is a widely known cosmetic component with antioxidant properties [72]. These proteins have a huge spectrum of application because of the eminent compatibility to human cells and simple biodegradability. Hydrolyzed collagen acts as a natural humectant with an excellent potential of becoming a harmless and useful biomaterial. It has been shown that supplementation and topical application of hydrolyzed proteins can be considered complementary in the improvement of general skin condition, participating in different mechanisms [73]. The review of sources and application of hydrolyzed collagen has been performed by León-López et al. [75]. The review on beneficial effects of food supplements based on hydrolyzed collagen for skin care has been conducted by Lupu et al. [76]. This review shows that bioactive peptides, such as collagen hydrolyzate, are among the most used ingredients for the development of nutraceuticals—food or food ingredients that have defined physiological effects. Numerous studies have demonstrated that peptides resulting from ingestion of collagen hydrolysate and detected in the bloodstream have chemotactic properties for skin fibroblasts, helping the skin restoration process. Poor stability of hydrolyzed fish collagen may hamper its application. For this reason, the use of liposomes as a vesicle has been studied as a potential mean to enhance the bioactivities and stability of such hydrolyzed collagen [77].

## 8. Collagen Modification by Cross-Linking

Collagen for cosmetic applications can be modified by a properly selected process of cross-linking. Low resistance to high temperature and enzymes dictates the necessity of structural stabilization of collagen. It is possible to distinguish physical, chemical, and biological methods applied for improvement of collagen structure. Chemical agents which are usually used for collagen crosslinking interact with amino- and carboxyl- groups of collagen, creating cross-links [78]. Nevertheless, some of them exhibit toxic properties. Extensively applied chemical cross-linking agents include glutaraldehyde (GA), genipin, 1-ethyl-3-(3-dimethylaminopropyl) carbodiimide (EDC) and N-hydroxysuccinimide (NHS), chitosan, dialdehyde starch. Cross-linking with glutaraldehyde is very efficient, at first it leads to the Schiff bases formations, then, after many subsequent reactions, to numerous diverse products. The research revealed that collagen cross-linked by GA becomes adequate in tissue engineering [79]. Various research groups tested genipin as a collagen structure stabilizer and tissue analogue [80]. Genipin and genipin scaffolds enhance cell proliferation and differentiation [81,82,83]. Research shows its potential in the endoscopic treatment of selected ulcers. Collagen gels ameliorated its deposition on ulcers, which enabled collagen penetration into submucosal layers. EDC-NHS enhances collagen stability to enzymes (for example collagenase). Furthermore, collagen stabilized by EDC-NHS in proper form is used as a specific system that delivers adequate ingredients [84]. Collagen cross-linked using EDC-NHS showed improved mechanical properties and good biocompatibility [85]. Dialdehyde starch (DAS) which is made from starch and periodic acid is the next cross-linking agent of collagen [86]. DAS characterizes itself with low toxicity, biodegradability, and exhibits antiviral properties [87,88]. Researchers designed the scaffold from materials like silk fibroin, collagen, and chitosan. The structure of this material has been stabilized by the process of cross-linking with the usage of DAS. Studies have shown that 3D material has an exceptional capacity to retain water due to numerous pores in its structure [89]. Due to the fact that the material was designed for bone tissue engineering, the compatibility to cells was confirmed. DAS has also been used for cross-linking of collagen/hyaluronic acid/chitosan material [90]. It did not exhibit any toxic properties and can be applied as a biomaterial in medicine and cosmetics. Chitosan comprises a linear polysaccharide which is created by the use of chitin with an alkaline agent [91]. Chitosan constitutes a molecule that is weakly soluble in water solution. Acidic medium ascends its solubility [92]. Collagen cross-linked by chitosan has its application in supporting chondrocyte as a scaffold [93,94,95]. Moreover, a collagen/chitosan matrix cross-linked by EDC-NHS and a 2-morpholinoethane sulfonic acid (MES) may be used as a matrix for artificial liver [96]. Contrarily, heating, drying, and irradiation constitute the physical methods of collagen cross-linking, which do not cause any potential harm to a patient’s tissue after material implantation [95]. The structure of collagen could be also stabilized by enzymes, for example, lysyl oxidase (LOX) [96]. Nowadays, the enzyme cross-linking process is conducted by microbial transglutaminase (MTG). MTG is used to meliorate the physical properties of protein-based materials. Gelatin-based hydrogels are stabilized by the cross-linking process using MTG [97,98]. The research has shown that MTG cross-linking process increases the material stability and mechanical resistance as well as the viability of fibroblasts [99]. It is worth to mention that using MTG does not cause any disturbance in the collagen structure [100,101]. Enzymatically-induced collagen cross-linking process not only improves the mechanical properties of the material but also shows fibroblasts cytocompatibility, which can be an interesting issue for tissue engineering [102]. Application of collagen biomaterials is still expanding from laboratories to applied medicine, contributing to a tremendous development in regenerative medicine and tissue engineering [103]. Although cross-linked collagen is rather rarely used in cosmetic applications, it can be still considered for preparation of beauty masks and wound dressing.

## 9. Collagen Blends for Cosmetic Applications

**Figure 4:**
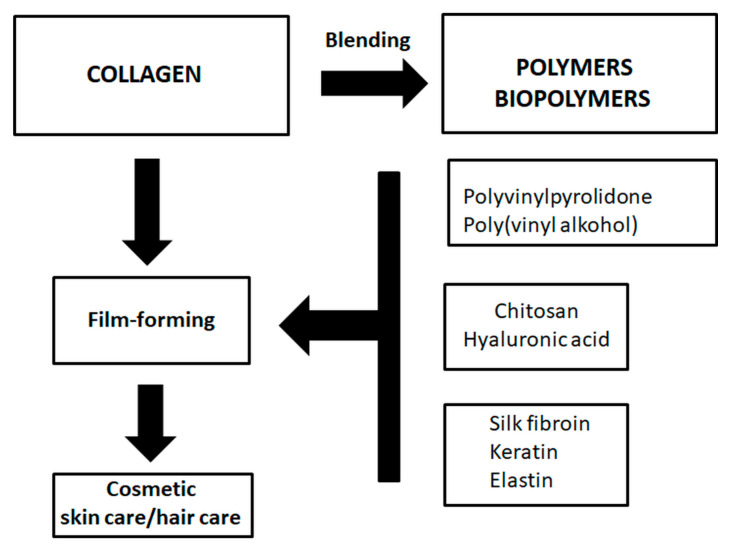
Polymers and biopolymers used for artificial blending with collagen.

Film-forming properties of collagen could be enhanced by binding collagen with other polymeric molecule and/or biopolymers [104,105,106,107,108,109,110,111,112,113,114,115,116,117,118,119,120,121,122,123]. Collagen and its film-forming properties can be modified by blending with polyvinylpyrrolidone (PVP) and polyvinyl alcohol (PVA) [104,105,106]. Our previous results showed that collagen and PVP mainly interact in the form of creating hydrogen bonds between collagen and PVP. Collagen acts as a donor of the hydrogen whereas PVP uses carbonyl group to create a bond. A proton-accepting carbonyl moiety in PVP as well as hydroxyl and amino groups in collagen are the main sides of formation of hydrogen bonds. Hydrogen bonds also represent the main issue in the interaction between collagen and PVA. Usually, the surface of the blend is enriched in one of the components of the blend and may have an influence on the film adhesion to the skin or hair. Surface free energy of the collagen/PVP blend is similar to the surface free energy of collagen. Film-forming properties of collagen solution can be modified by the addition of chitosan [107,108,109,110,111,112,113]. Some research showed the possibility of tailoring certain properties by changing component ratios, since different interactions occurred between the two macromolecules. Collagen film can be modified by the addition of elastin [114], keratin [115], silk fibroin [116,117,118,119], and hyaluronic acid [120,121,122,123,124]. In Figure 4 polymers and biopolymers used for artificial blending with collagen have been shown [104,105,106,107,108,109,110,111,112,113,114,115,116,117,118,119,120,121,122,123,124,125].

After the addition of the second polymer and/or biopolymer to collagen, the obtained films showed modified mechanical properties, modified surface roughness and modified wettability. All of these film properties are very important in cosmetic applications [125]. Collagen blends with other polymers and/or biopolymers also reveal some potential for biomedical applications, for example for artificial skin, bones, membranes, hydrogels, and many other products [119,120,121,122].

## 10. The Comparison of the Existing Knowledge in the Field of Collagen Application in Cosmetics

In the scientific literature, there are many papers regarding collagen; however, when comparing the results and existing knowledge about applications of collagen in cosmetics, the small amount of records in common databases constitutes the major problem. Although collagen is extensively used in cosmetics, the results are not always published, as some research may be conducted by cosmetic companies which usually do not publish new achievements in scientific papers. Very often, the results generated in cosmetic companies are patented or simply used to improve cosmetic products.

According to the Scopus Data Base, approximately 297,053 papers have been published in which the word “collagen” appears in a title, keyword, abstract (approximately 63,192 in article title). These data show that collagen is widely studied within the scientific community. However, only approximately 35 papers can be found in Scopus Data Base when we use together the word “collagen” and “cosmetic” in an article title in order to find results. When the search takes titles, abstracts, and keywords into account, the results are more vast—approximately 2368 documents. These data were collected in September 2020. It is possible that there are also reports not provided by Scopus, so they are not easily accessible.

It is known that the collagen extraction method (acid-soluble, pepsin-soluble, electrodialysis, ultrasound, isoelectric precipitation) may directly influence its properties [126], so sometimes it is not easy to compare results regarding collagen properties obtained in different laboratories and its cosmetic applications. Undeniably, fish collagen is widely used in cosmetic applications; however, its properties may differ depending on the kind of fish and methods of collagen extraction. Alternative and safe sources of collagen have been studied by Tziveleka et al. [127]. As a source of collagen, the marine demosponges Axinella cannabina and Suberites carnosus, collected from the Aegean and the Ionian Seas, respectively, were studied. It can be expected that many other sources of collagen will be proposed in the future. Although fish collagen and mammalian collagen have different amino acids compositions, both of these kinds of collagen can be used in cosmetic applications [128,129]. Fish collagen is usually less thermally stable than mammalian collagen due to the small amount of hydroxyproline and a bigger amount of serine, threonine, and methionine. Nevertheless, the abundance of fish collagen and the safety of its application determine the common use of this kind of collagen. It should also be mentioned that non-animal collagens as a new option for cosmetic applications have been proposed as alternatives to animal collagens for inclusion in cosmetic formulations [130].

## 11. Conclusions

Collagen is widely used in cosmetic preparations. The role of collagen is to increase skin hydration and prevent skin ageing. Film-forming properties of collagen materials can be modified by collagen cross-linking and/or blending of collagen with other proteins and polysaccharides. Future research directions on collagen application for cosmetic purposes may be focused on increasing the denaturation temperature of several types of collagen extracted from fish species. Such an increase of denaturation temperature may expand collagen application not only in cosmetic fields. Collagen will be used without any doubt for effective rejuvenating treatments of an ageing population, as the ageing of the skin is a process with very direct effects on daily life, as well as the psychological and social well-being of an individual. The more youthful appearance will most likely have beneficial effects for the whole organism and would help to keep the proper position in society.
